# When the heat is on, HYL1 steps in: Regulating miRNA biogenesis for plant thermotolerance

**DOI:** 10.1093/plcell/koaf111

**Published:** 2025-05-06

**Authors:** Regina Mencia

**Affiliations:** Assistant Features Editor, The Plant Cell, American Society of Plant Biologists; Instituto de Agrobiotecnología del Litoral (CONICET-UNL), Cátedra de Biología Celular y Molecular, Facultad de Bioquímica y Ciencias Biológicas, Universidad Nacional del Litoral, Santa Fe 3000, Argentina

Changes in environmental temperature are a major source of stress for plants. Plants have evolved various adaptive strategies that combat heat stress (HS) and mitigate potential negative effects on development and productivity. One such strategy involves microRNAs (miRNAs), small single-stranded RNAs, typically 21 nucleotides long, that play key regulatory roles, primarily through post-transcriptional gene silencing. In this process, miRNAs, once loaded into a protein complex, recognize target transcripts, leading to their cleavage and subsequent degradation (Huntzinger and Izaurralde 2011).

While specific miRNAs have been linked to HS responses, **Yiming Cao, Jiaxin Zhang, and colleagues (Cao et al. 2025)** set out to explore the broader, global role of miRNAs in plant adaptation to HS, using Arabidopsis (*A. thaliana*) as a model organism. Their findings reveal that most miRNAs are upregulated under HS, leading to the downregulation of their target genes. However, an intriguing observation emerged: pri-miRNA (hairpin-folded primary miRNA transcript) levels did not follow the same pattern.

MicroRNA synthesis is a multi-step process. In the nucleus, the core microprocessor complex comprising DICER-LIKE1 (DCL1), SERRATE (SE), and HYPONASTIC LEAVES 1 (HYL1) recognizes pri-miRNAs and processes them into mature miRNAs (Mencia et al. 2023). The observed discrepancy between pri-miRNA and mature miRNA levels suggests that HS may influence the global miRNA biogenesis pathway rather than just individual miRNAs.

A closer look at the miRNA biogenesis pathway revealed a surprising finding: although overall HYL1 levels decreased under HS, its nuclear localization increased. The routing of HYL1 to the nucleus led to greater binding to pri-miRNAs in HS-treated plants, which could explain the higher miRNA accumulation due to increased microprocessor activity. Furthermore, mutants with defects in the microprocessor displayed greater sensitivity to HS than wild-type plants, underscoring the crucial role of miRNA biogenesis in HS responses.

It was previously shown that nuclear localization of HYL1 is orchestrated by its phosphorylation state (Gonzalo et al. 2024). This led the authors to investigate whether HS could affect HYL1 phosphorylation, thereby triggering increased miRNA biogenesis. Analysis of mutant plants carrying a nonphosphorylated version of HYL1 revealed reduced nuclear localization, lower miRNA induction, and increased sensitivity to HS, further supporting the central role of HYL1 phosphorylation in the HS response.

HYL1 phosphorylation is regulated by the dynamic balance between the phosphatase CPL1 and the kinase MPK3 (Gonzalo et al. 2024). In their study, Cao et al. (2025) examined the interaction between CPL1 and MPK3, determining that the 2 proteins physically interact. Additionally, they found that MPK3 phosphorylates CPL1, regulating its stability. Interestingly, mutants lacking CPL1 exhibited increased MPK3 kinase activity, suggesting a reciprocal regulatory mechanism between these 2 proteins (Figure). Moreover, when analyzing mutant plants lacking MPK3, they observed increased nuclear accumulation of HYL1, higher miRNA abundance, and enhanced tolerance to HS. Overall, the work by Cao et al. provides valuable insights into the role of miRNA biogenesis in HS adaptation, shedding light on the regulation of HYL1 and its contribution to plant stress responses.

**Figure.**
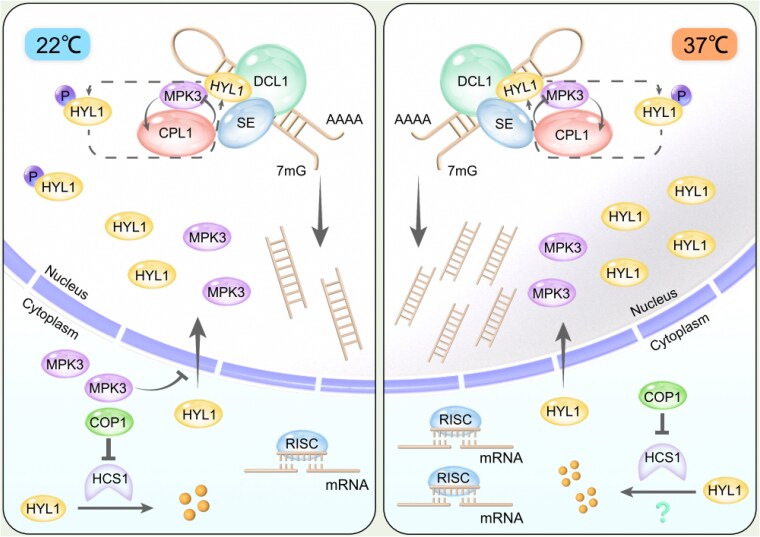
Under normal conditions (22 °C) MPK3 inhibits HYL1 nuclear translocation, while MPK3 and CPL1 interact in the nucleus to regulate HYL1 phosphorylation. Under heat stress (37 °C), HYL1 accumulates in the nucleus, increasing its interaction with pri-miRNAs and promoting miRNA biogenesis. Adapted from Cao et al. (2025), Figure 10.

## Recent related articles in *The Plant Cell*

- Li et al. (2023) demonstrate that HS induces the miR165/166–PHB regulatory module, which modulates HSFA1, controlling HS responses in *Arabidopsis thaliana* through transcriptional reprogramming and antagonistic interactions between PHB and HSFA1s.
- Field et al. (2023) review recent advances in understanding biomolecular condensates in plant development, examining their potential roles throughout the plant life cycle, their molecular functions, and how they respond to environmental changes.
- Pan et al. (2024) revealed that the SMXL4/SMXL5–HSFA2 regulatory module modulates plant thermotolerance by repressing *HSFA2* transcription and antagonizing HSFA1 activity, while HSFA2, in turn, induces *SMXL4* and *SMXL5* expression during heat stress recovery, establishing a feedback mechanism in *Arabidopsis thaliana*.
- Park et al. (2023) showed that HYL1 mediates the ABA-induced recruitment of the HOS15-HDA9 complex to MIRNA loci, linking pri-miRNA-structure recognition to the transcriptional repression of miRNA biogenesis.

## Dive Curated Terms

The following phenotypic, genotypic, and functional terms are of significance to the work described in this paper:

CPL1 Gramene: AT4G21670
CPL1 Araport: AT4G21670

PHB Gramene: AT2G34710
PHB Araport: AT2G34710

MPK3 Gramene: AT3G45640
MPK3 Araport: AT3G45640

HSFA2 Gramene: AT2G26150
HSFA2 Araport: AT2G26150

HYL1 Gramene: AT1G09700
HYL1 Araport: AT1G09700

DCL1 Gramene: AT1G01040
DCL1 Araport: AT1G01040

HSFA1 Gramene: At1g32330, At3g02990, At4g17750, At5g16820
HSFA1 Araport: At1g32330, At3g02990, At4g17750, At5g16820

SMXL4 Gramene: AT4G29920
SMXL4 Araport: AT4G29920

SMXL5 Gramene: AT5G57130
SMXL5 Araport: AT5G57130
